# Synthesis, Antioxidant, Cytotoxicity, Induce Apoptosis Investigation and Docking Study of New Halogenated Dihydropyrano[3,2-*b*]Chromene-3-Carbonitrile Derivatives on MCF-7 Breast Cancer Cell Line

**DOI:** 10.5812/ijpr-132932

**Published:** 2023-04-18

**Authors:** Touba Eslaminejad, Ehsan Faghih Mirzaei, Mehdi Abaszadeh

**Affiliations:** 1 Pharmaceutics Research Center, Institute of Neuropharmacology, Kerman University of Medical Sciences, Kerman, Iran; 2 Department of Medicinal Chemistry, Faculty of Pharmacy, Kerman University of Medical Sciences, Kerman, Iran

**Keywords:** 6-Chloro-3-Hydroxychromone, 6-Bromo-3-Hydroxychromone, Cytotoxicity, Cancer Cell Line, Chromone Derivatives, Docking Study

## Abstract

**Background:**

Chromene derivatives showed numerous biological activities. In the current study, the antioxidant, cytotoxicity, and apoptosis properties of halogenated dihydropyrano[3,2-*b*]chromene-3-carbonitrile derivatives (HDCCD) on MCF-7 cell line have been examined.

**Objectives:**

This study's principal point was synthesizing new halogenated pyranochromene derivatives and assessing their cytotoxic effects and apoptosis potential on MCF-7 breast cancer cell line by flow cytometry.

**Methods:**

Initially, 6-chloro- and 6-bromo-3-hydroxychromone compounds were prepared. In the next step, a series of HDCCD were synthesized by a one-pot three-component reaction of these two compounds, aromatic aldehydes, and malononitrile, in the presence of triethylamine in EtOH at reflux conditions. These compounds were fully characterized by standard spectroscopic techniques (IR, ^1^H, and ^13^C NMR) and elemental analyses. The potential of the antioxidant activity was determined by using ferric reducing antioxidant power assay (FRAP). 3-(4,5-Dimethylthiazol-2-yl)-2,5-diphenyltetrazolium bromide (MTT) assay and lactate dehydrogenase (LDH) were used to evaluate metabolic activity. The nitric oxide (NO) and malondialdehyde (MDA) biomarkers of the exposed cells were evaluated on the cells and their supernatant. To quantify apoptotic death of MCF-7 breast cancer cells treated by the compounds at their IC_50_ concentrations, Annexin V-FITC apoptosis detection kit was utilized. Molecular docking of compounds (6a-j) into the Cyclin-dependent kinase 6 (PDB code: 4EZ5) was carried out, and the probable binding mode of compounds 6e and 6j was determined.

**Results:**

A dose-response relationship was seen in all the compounds. Most of them induced cytotoxic effects on the cells. Nitrite concentration of the culture media of the cells was decreased compared to the control. Malondialdehyde levels of the cells were below the range of the control by the addition of 6b, 6d, 6e, 6f, and 6g compounds on the cells, while the addition of the 6a, 6c, 6h, 6i, and 6j compounds increased the MDA level compared to the control. Flow cytometric analysis showed that most of the exposed cells were in the early and late apoptotic stage, and a few of them were in the necrotic stage.

**Conclusions:**

It could be concluded that HDCCD (6a-j) was toxic and caused death in the cells by apoptosis. The compounds have lipophilic characteristics, so they can easily pass the cell membrane. As confirmed by LDH results, it can be concluded that the cytotoxicity is connected with apoptosis rather than necrosis, endorsed by flowcytometry analysis afterward.

## 1. Background

Compounds in which benzene and a 4-pyrone ring are fused are called 4*H*-1-benzopyran-4-one, 4*H*-chromen-4-one, or chromone. Chromones are a gathering of natural compounds omnipresent in nature, particularly in plants (1, 2). They are present in the human diet and show less toxicity to mammalian cells. They are found in the core structure of various flavonoids; for example, 3-hydroxyflavone is a compound with a phenyl group in the 2-position and a hydroxyl group in the 3-position in the pyrone ring of chromone scaffold. Chromone derivatives display a wide range of biological activities, including anti-carcinogenic effects (3), anti-HIV (4), antibacterial (5), anti-obesity and antidiabetic (6), cardiovascular risk reduction (7), antifungal (8), and antiviral activity (9). They are also used as cognitive enhancers for treating neurodegenerative diseases (10). The nitrovinyl side chain connected to an aromatic ring is a compelling pharmacophore for developing modern pro-apoptotic agents (11). Due to their biological activities in the human body, there has been considerable interest in the development and pharmacology of these heterocyclic compounds (12). Chromenes as heterocyclic scaffolds in O-heterocycles class represent a significant biological activity (13). Natural chromenes include stilbenoids, coumarins, and flavonoids; stilbenoids such as resveratrol, pterostilbene, gentle, and piceatannol; all show biological activity. According to the literature, developing efficient and convenient methods for synthesizing chromene derivatives is necessary.

## 2. Objectives

In continuing our previous work on the dihydropyrano[3,2-*b*]chromene derivatives, the three-component reaction of 6-chloro- and 6-bromo-3-hydroxychromone (3a, b), aromatic aldehydes (4a-j) and malononitrile (5) in EtOH, at reflux and in the presence of triethylamine as the base was used to prepare halogenated dihydropyrano[3,2-*b*]chromene-3-carbonitrile derivatives (HDCCD) compounds (6a-j). Thereafter, the antioxidant, cytotoxic, and apoptosis effects of these compounds on the MCF-7 breast cancer cell line were investigated.

## 3. Methods

All used reagents and chemicals were prepared from commercial sources and applied with no further purification. Determination of the melting points was done on the Electrothermal-9100 apparatus without correction. Using KBr pallets, IR spectra were recorded on a Bruker FTIRspectrophotometer (Alpha model). ^13^C NMR (75 MHz) and ^1^H NMR (300 MHz) spectra were recorded on a Bruker AVANCE III 300 MHz spectrometer in dimethyl sulfoxide (DMSO)-*d*_6_ and TMS was an internal standard. Coupling constants (*J*) are given in Hz and chemical shifts (*δ*) are expressed in parts per million (ppm). Thin layer chromatography (TLC) was used to monitor reactions on the Aluminium-backed silica gel sheets (GF254) and were observed in UV light (254 nm). Elemental analyses were employed utilizing a Heraeus CHN-O-Rapid analyzer. Dulbecco's modified eagle's medium F12 (DMEM-F12), fetal bovine serum (FBS), penicillin-streptomycin (100 μg/mL), and phosphate-buffered saline (PBS), trypan blue dye solution, trypsin- EDTA solution, and DMSO, 3-(4,5-Dimethylthiazol-2-yl)-2,5-diphenyltetrazolium bromide (MTT reagent), FeCl_3_, 2,4,6-Tris(2-pyridyl)-s-triazine (TPTZ), acetate buffer as ferric reducing antioxidant power assay (FRAP) reagent and thiobarbituric acid (TBA), N-(1-Naphthyl) -ethylenediamine dihydrochloride (Griess reagent) and Annexin V-FITC apoptosis detection kit were obtained from the Sigma-Aldrich^®^ Company, Germany.

### 3.1. Preparation of 6-Chloro- and 6-Bromo-3-Hydroxychromone (3a, b)

**Figure 1.**
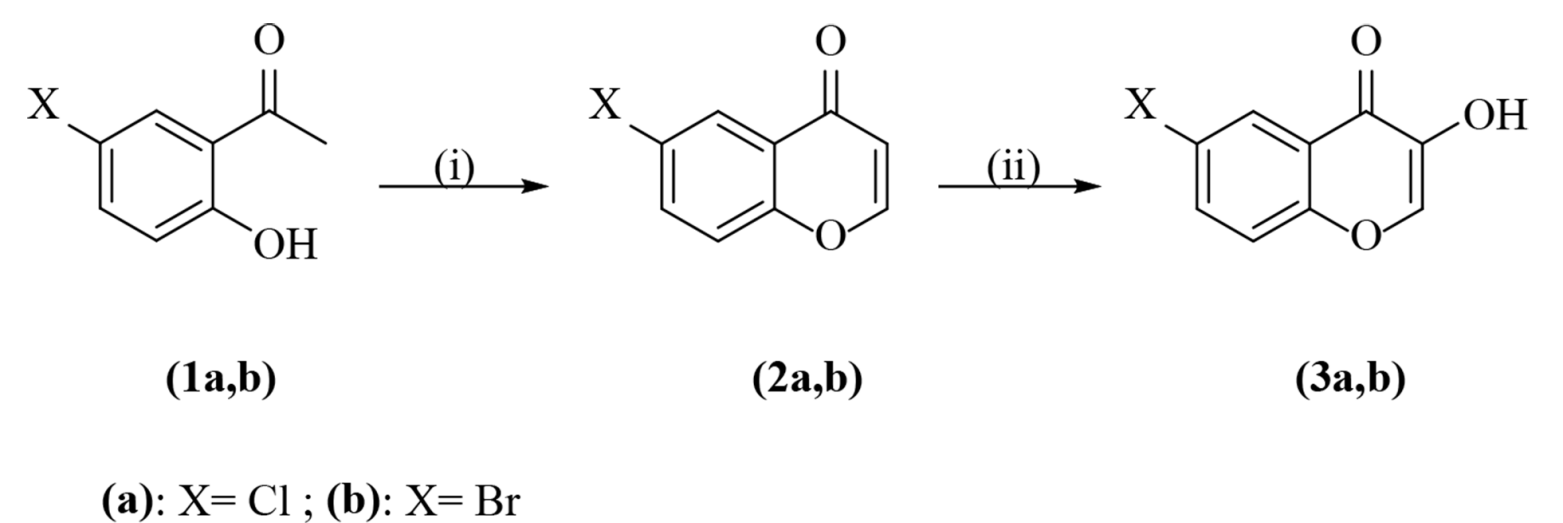
Synthesis of 6-chloro-, and 6-bromo-3-hydroxychromone. Reagents and conditions: (i): (1) N,N-dimethylformamide-dimethylacetal (DMFDMA)/MW; (2) HCl (con.), CH_2_Cl_2_, reflux; (ii): (1) H_2_O_2_, NaOH, CH_2_Cl_2_, ice-bath; (2) HCl (con.), reflux.

These compounds were prepared according to the literature procedures presented by Spadafora et al. and represented in Figure 1 (14). The isolated products were crystallized from ethanol.

**6-Chloro-3-Hydroxychromone (3a): **White powder; yield: 91%; mp 218 - 219°C (lit. (15) 216°C); IR (KBr, cm^-1^) ν_max_: 3296 (OH), 1629 (C=O), 1605 (C=C); ^1^H NMR (300 MHz, DMSO-*d*_6_) δ_ppm_: 9.36 (br, 1H, OH), 8.26 (s, 1H, CH), 8.05 (d, J = 3 Hz, 1H, ArH), 7.80 (dd, *J* = 9 Hz, 3 Hz, 1H, ArH), 7.71 (d, *J* = 9 Hz, 1H, ArH); ^13^C NMR (75 MHz, DMSO-*d*_6_) δ_ppm_: 172.10 (C=O), 154.26 (C3), 142.45 (C2), 141.73, 133,74, 129.49, 124.29, 124.25, 121.44; Anal. calcd. for C_9_H_5_ClO_3_: C, 54.99; H, 2.56%. Found: C, 54.83; H, 2.41%.

**6-Bromo-3-Hydroxychromone (3b): **Pale yellow powder; yield: 88%; mp 205 - 206°C; IR (KBr, cm^-1^) ν_max_: 3101 (OH), 1623 (C=O), 1601 (C=C); ^1^H NMR (300 MHz, DMSO-*d*_6_) *δ*_ppm_: 9.35 (br, 1H, OH), 8.27 (s, 1H, CH), 8.17 (d, *J* = 3 Hz, 1H, ArH), 7.89 (dd, *J* = 9 Hz, 3 Hz, 1H, ArH), 7.62 (d, *J* = 9 Hz, 1H, ArH); ^13^C NMR (75 MHz, DMSO-*d*_6_) *δ*_ppm_: 171.96 (C=O), 154.60 (C3), 142.50 (C2), 141.70, 136.38, 127.40, 124.72, 121.59, 117.35; Anal. calcd. for C_9_H_5_BrO_3_: C, 44.85; H, 2.09%. Found: C, 44.64; H, 1.96%.

### 3.2. General Procedure for the Preparation of Halogenated Dihydropyrano[3,2-b]Chromene-3-Carbonitrile Derivatives (6a-j)

A mixture of 6-chloro- or 6-bromo-3-hydroxychromone (3a, b) (2 mmol), aromatic aldehydes (4a-j) (2 mmol), and malononitrile (5) (2.1 mmol), and three drops of triethylamine in ethanol (10 mL) were added to a 50 mL round-bottomed flask equipped with a magnetic blending bar and a reflux condenser. It was stirred and refluxed for 1 h. The progress of the reaction was observed by TLC using hexane/ethyl acetate as an eluent. After completion of the reaction, the mixture was cooled, and the obtained crude product was filtered, washed with ethanol, and crystallized from ethanol to give the pure solid sample for analysis.

**2-Amino-8-Chloro-10-Oxo-4-Phenyl-4,10- Dihydropyrano[3,2-b]Chromene-3-Carbonitrile (6a):** Cream powder; yield: 89%; mp 245 - 246°C; IR (KBr, cm^-1^) ν_max_: 3440, 3330 (NH_2_), 3027 (CH, aromatic), 2193 (CN), 1657 (CO), 1632 (C=C); ^1^H NMR (300 MHz, DMSO-*d*_6_) *δ*_ppm_: 8.00 (d, *J* = 3 Hz, 1H, ArH), 7.75 (dd, *J* = 9 Hz, 3 Hz, 1H, ArH), 7.55 (d, *J* = 9 Hz, 1H, ArH), 7.46 - 7.34 (m, 7H, NH_2_, ArH), 4.96 (s, 1H, CH); ^13^C NMR (75 MHz, DMSO-*d*_6_) *δ*_ppm_: 167.99 (C=O), 159.69 (C-2), 153.54, 150.78, 141.24, 134.71, 133.86, 130.41, 129.46, 128.44, 124.78, 124.64, 121.12, 119.75 (CN), 56.05 (C-3), 41.44 (C-4); Anal. calcd. for C_19_H_11_ClN_2_O_3_: C, 65.06; H, 3.16; N, 7.99%. Found: C, 65.13; H, 3.02; N, 7.81%.

**2-Amino-8-Chloro-4-(4-Chlorophenyl)-10-Oxo-4,10- Dihydropyrano[3,2-b]Chromene-3-Carbonitrile (6b):** Yellow powder; yield: 93%; mp 224 - 225°C; IR (KBr, cm^-1^) ν_max_: 3404, 3316 (NH_2_), 3041 (CH, aromatic), 2968 (CH, aliphatic), 2202 (CN), 1651 (C=O), 1633 (C=C); ^1^H NMR (300 MHz, DMSO-*d*_6_) *δ*_ppm_: 8.02 (d, *J* = 3 Hz, 1H, ArH), 7.78 (dd, *J* = 9 Hz, 3 Hz, 1H, ArH), 7.58 (d, *J* = 9 Hz, 1H, ArH), 7.49 - 7.42 (m, 4H, ArH), 7.37 (brs, 2H, NH_2_), 5.04 (s, 1H, CH); ^13^C NMR (75 MHz, DMSO-*d*_6_) *δ*_ppm_: 168.05 (C=O), 159.68 (C-2), 153.59, 150.18, 140.16, 134.78, 133.95, 133.12, 130.44, 129.43, 124.82, 124.66, 121.17, 119.62 (CN), 55.67 (C-3), 40.82 (C-4); Anal. calcd. for C_19_H_10_Cl_2_N_2_O_3_: C, 59.24; H, 2.62; N, 7.27%. Found: C, 59.11; H, 2.47; N, 7.29%.

**2-Amino-8-Chloro-10-Oxo-4-(p-Tolyl)-4,10- Dihydropyrano[3,2-b]Chromene-3-Carbonitrile (6c):** Pale yellow powder; yield: 86%; mp 217 - 218°C; IR (KBr, cm^-1^) ν_max_: 3409, 3323 (NH_2_), 3026 (CH, aromatic), 2970 (CH, aliphatic), 2199 (CN), 1648 (C=O), 1632 (C=C); ^1^H NMR (300 MHz, DMSO-*d*_6_) *δ*_ppm_: 8.00 (d, *J* = 3 Hz, 1H, ArH), 7.76 (dd, *J* = 9 Hz, 3 Hz, 1H, ArH), 7.56 (d, *J* = 9 Hz, 1H, ArH), 7.30 - 7.19 (m, 6H, NH_2_, ArH), 4.90 (s, 1H, CH), 2.29 (s, 3H, CH_3_); ^13^C NMR (75 MHz, DMSO-*d*_6_) *δ*_ppm_: 168.00 (C=O), 159.62 (C-2), 153.54, 150.98, 138.32, 137.72, 134.71, 133.74, 131.14, 130.61, 130.40, 130.00, 128.33, 124.77, 124.64, 121.13, 119.76 (CN), 56.29 (C-3), 41.09 (C-4), 21.13 (CH_3_); Anal. calcd. for C_20_H_13_ClN_2_O_3_: C, 65.85; H, 3.59; N, 7.68%. Found: C, 65.69; H, 3.61; N, 7.51%.

**2-Amino-4-(4-Bromophenyl)-8-Chloro-10-Oxo-4,10- Dihydropyrano[3,2-b]Chromene-3-Carbonitrile (6d):** Cream powder; yield: 91%; mp 235 - 236°C; IR (KBr, cm^-1^) ν_max_: 3368, 3307 (NH_2_), 3044 (CH, aromatic), 2960 (CH, aliphatic), 2202 (CN), 1654 (C=O), 1633 (C=C); ^1^H NMR (300 MHz, DMSO-*d*_6_) *δ*_ppm_: 8.02 (d, *J* = 3 Hz, 1H, ArH), 7.79 (dd, *J* = 9 Hz, 3 Hz, 1H, ArH), 7.62 - 7.58 (m, 3H, ArH), 7.39 - 7.37 (m, 4H, NH_2_, ArH), 5.02 (s, 1H, CH); ^13^C NMR (75 MHz, DMSO-*d*_6_) *δ*_ppm_: 168.05 (C=O), 159.68 (C-2), 153.59, 150.12, 140.59, 134.78, 133.95, 132.35, 130.79, 130.44, 124.82, 124.66, 121.70, 121.17, 119.62 (CN), 55.60 (C-3), 40.82 (C-4); Anal. calcd. for C_19_H_10_BrClN_2_O_3_: C, 53.11; H, 2.35; N, 6.52%. Found: C, 53.13; H, 2.19; N, 6.39%.

**2-Amino-8-Chloro-4-(4-Methoxyphenyl)-10-oxo-4,10 -Dihydropyrano[3,2-b]Chromene-3-Carbonitrile (6e):** Yellow powder; yield: 90%; mp 204 - 205°C; IR (KBr, cm^-1^) ν_max_: 3409, 3323 (NH_2_), 3026 (CH, aromatic), 2970 (CH, aliphatic), 2199 (CN), 1648 (C=O), 1632 (C=C); ^1^H NMR (300 MHz, DMSO-*d*_6_) *δ*_ppm_: 8.00 (d, *J* = 3 Hz, 1H, ArH), 7.75 (dd, *J* = 9 Hz, 3 Hz, 1H, ArH), 7.55 (d, *J* = 9 Hz, 1H, ArH), 7.32 - 7.29 (m, 4H, NH_2_, ArH), 6.95 (d, *J* = 9 Hz, 2H, ArH), 4.89 (s, 1H, CH), 3.75 (s, 3H, OCH_3_); ^13^C NMR (75 MHz, DMSO-*d*_6_) *δ*_ppm_: 168.00 (C=O), 159.58 (C-2), 159.38, 153.54, 151.07, 134.69, 133.64, 133.24, 130,38, 129.58, 124.75, 124.64, 124.24, 121.11, 119.80 (CN), 115.64, 114.78, 56.29 (C-3), 55.57 (OCH_3_), 40.81 (C-4); Anal. calcd. for C_20_H_13_ClN_2_O_4_: C, 63.09; H, 3.44; N, 7.36%. Found: C, 63.11; H, 3.49; N, 7.21%.

**2-Amino-8-Bromo-10-Oxo-4-Phenyl-4,10- Dihydropyrano[3,2-b]Chromene-3-Carbonitrile (6f):** White powder; yield: 85%; mp 272 - 273°C; IR (KBr, cm^-1^) ν_max_: 3440, 3329 (NH_2_), 3027 (CH, aromatic), 2916 (CH, aliphatic), 2193 (CN), 1657 (C=O), 1632 (C=C); ^1^H NMR (300 MHz, DMSO-*d*_6_) *δ*_ppm_: 8.15 (d, *J* = 3 Hz, 1H, ArH), 7.87 (dd, *J* = 9 Hz, 3 Hz, 1H, ArH), 7.51 (d, *J* = 9 Hz, 1H, ArH), 7.44 - 7.32 (m, 7H, NH_2_, ArH), 4.96 (s, 1H, CH); ^13^C NMR (75 MHz, DMSO-*d*_6_) *δ*_ppm_: 167.88 (C=O), 159.66 (C-2), 153.94, 150.79, 141.23, 137.44, 133.88, 129.45, 128.42, 127.76, 125.19, 121.32, 119.70 (CN), 118.29, 56.06 (C-3), 41.42 (C-4); Anal. calcd. for C_19_H_11_BrN_2_O_3_: C, 57.74; H, 2.81; N, 7.09%. Found: C, 57.49; H, 2.65; N, 7.00%.

**2-Amino-8-Bromo-4-(4-Chlorophenyl)-10-Oxo-4,10- Dihydropyrano[3,2-b]Chromene-3-Carbonitrile (6g):** Pale yellow powder; yield: 91%; mp 243 - 244°C; IR (KBr, cm^-1^) ν_max_: 3399, 3317 (NH_2_), 3088 (CH, aromatic), 2969 (CH, aliphatic), 2201 (CN), 1650 (C=O), 1635 (C=C); ^1^H NMR (300 MHz, DMSO-*d*_6_) *δ*_ppm_: 8.13 (d, *J* = 3 Hz, 1H, ArH), 7.87 (dd, *J* = 9 Hz, 3 Hz, 1H, ArH), 7.51 - 7.42 (m, 5H, ArH), 7.37 (brs, 2H, NH_2_), 5.03 (s, 1H, CH); ^13^C NMR (75 MHz, DMSO-*d*_6_) *δ*_ppm_: 167.91 (C=O), 159.68 (C-2), 153.95, 150.17, 140.14, 137.45, 133.96, 133.13, 130.44, 129.42, 127.76, 125.19, 121.30, 119.62 (CN), 118.32, 55.66 (C-3), 40.82 (C-4); Anal. calcd. for C_19_H_10_BrClN_2_O_3_: C, 53.11; H, 2.35; N, 6.52%. Found: C, 52.90; H, 2.39; N, 6.37%.

**2-Amino-8-Bromo-10-Oxo-4-(p-Tolyl)-4,10- Dihydropyrano[3,2-b]Chromene-3-Carbonitrile (6h):** Yellow powder; yield: 85%; mp 267 - 268°C; IR (KBr, cm^-1^) ν_max_: 3404, 3320 (NH_2_), 3087 (CH, aromatic), 2919 (CH, aliphatic), 2198 (CN), 1648 (C=O), 1633 (C=C); ^1^H NMR (300 MHz, DMSO-*d*_6_) *δ*_ppm_: 8.13 (d, *J* = 3 Hz, 1H, ArH), 7.85 (dd, *J* = 9 Hz, 3 Hz, 1H, ArH), 7.47 (d, *J* = 9 Hz, 1H, ArH), 7.30 - 7.19 (m, 6H, NH_2_, ArH), 4.90 (s, 1H, CH), 2.29 (s, 3H, CH_3_); ^13^C NMR (75 MHz, DMSO-*d*_6_) *δ*_ppm_: 167.87 (C=O), 159.61 (C-2), 153.92, 150.97, 138.31, 137.72, 137.41, 133.76, 131.14, 130.60, 130.00, 128.33, 127.75, 125.15, 121.28, 119.76 (CN), 118.28, 56.16 (C-3), 41.10 (C-4), 21.13 (CH_3_); Anal. calcd. for C_20_H_13_BrN_2_O_3_: C, 58.70; H, 3.20; N, 6.85%. Found: C, 58.67; H, 3.22; N, 6.69%.

**2-Amino-8-Bromo-4-(4-Bromophenyl)-10-Oxo-4,10 -Dihydropyrano[3,2-b]Chromene-3-Carbonitrile (6i):** Yellow powder; yield: 89%; mp 239 - 240°C; IR (KBr, cm^-1^) ν_max_: 3396, 3316 (NH_2_), 3088 (CH, aromatic), 2919 (CH, aliphatic), 2200 (CN), 1650 (C=O), 1635 (C=C); ^1^H NMR (300 MHz, DMSO-*d*_6_) *δ*_ppm_: 8.13 (d, *J* = 3 Hz, 1H, ArH), 7.87 (dd, *J* = 9 Hz, 3 Hz, 1H, ArH), 7.61 (d, *J* = 9 Hz, 2H, ArH), 7.49 (d, *J* = 9 Hz, 1H, ArH), 7.39 - 7.36 (m, 4H, NH_2_, ArH), 5.01 (s, 1H, CH); ^13^C NMR (75 MHz, DMSO-*d*_6_) *δ*_ppm_: 167.91 (C=O), 159.68 (C-2), 153.95, 150.11, 140.57, 137.46, 133.96, 133.13, 132.59, 132.34, 130.79, 127.76, 125.19, 121.71, 121.31, 119.62 (CN), 118.32, 55.58 (C-3), 40.83 (C-4); Anal. calcd. for C_19_H_10_Br_2_N_2_O_3_: C, 48.13; H, 2.13; N, 5.91%. Found: C, 48.17; H, 1.98; N, 5.63%.

**2-Amino-8-Bromo-4-(4-Methoxyphenyl)-10-Oxo-4, 10-Dihydropyrano[3,2-b]Chromene-3-Carbonitrile (6j):** Orange powder; yield: 88%; mp 212 - 213°C; IR (KBr, cm^-1^) ν_max_: 3401, 3320 (NH_2_), 3008 (CH, aromatic), 2927 (CH, aliphatic), 2199 (CN), 1647 (C=O), 1634 (C=C); ^1^H NMR (300 MHz, DMSO-*d*_6_) *δ*_ppm_: 8.13 (d, *J* = 3 Hz, 1H, ArH), 7.87 (dd, *J* = 9 Hz, 3 Hz, 1H, ArH), 7.50 (d, *J* = 9 Hz, 1H, ArH), 7.32 - 7.29 (m, 4H, NH_2_, ArH), 6.95 (d, *J* = 9 Hz, 2H, ArH), 4.89 (s, 1H, CH), 3.75 (s, 3H, OCH_3_); ^13^C NMR (75 MHz, DMSO-*d*_6_) *δ*_ppm_: 167.88 (C=O), 159.57 (C-2), 159.38, 153.93, 151.08, 137.66, 133.83, 133.67, 133.23, 129.59, 127.76, 125.16, 121.29, 119.80 (CN), 118.24, 115.65, 114.78, 56.29 (C-3), 55.58 (OCH_3_), 40.82 (C-4); Anal. calcd. for C_20_H_13_BrN_2_O_4_: C, 56.49; H, 3.08; N, 6.59%. Found: C, 56.33; H, 3.11; N, 6.43%.

### 3.3. Antioxidant Activity

The potential of the antioxidant activity of the HDCCD (6a-j) was determined by using iron chelating; FRAP assay (16). The FRAP reagent was made from a 1:1:10 mixture of three solutions: 20 mM FeCl_3_, 10 mM TPTZ, and 0.3 M acetate buffer adjusted at pH 3.6. The extinction coefficient (ε) for Fe (II) was calculated from a standard curve using a 2 mmol/L FeSO_4_ solution (0.56 mg/mL). In the dark, 0.5 mL of the HDCCD (6a-j) at different concentrations (0.01 - 100 mg/L) was mixed with 1 mL of 0.2 M phosphate buffer (pH 6.6), and 1 mL of 1% (w/v) potassium ferricyanide and the prepared mixture was then incubated at 50°C for 20 min. Trichloroacetic acid (1 mL, 10%) was subsequently added to the reaction mixture and centrifuged at 3000 rpm for 10 min. The obtained supernatant (1.5 mL) was then mixed with an equal volume of distilled water and 0.1 mL of ferric chloride (0.1%) and consequently incubated at room temperature for 10 min. The absorbance of the samples was then measured at 700 nm by a multimode microplate reader. Negative control was conducted the same as treatments except that distilled water was used instead of the sample. FeSO_4_ was applied as a positive control. Three replicates of each experiment were performed, and the mean of the obtained results was reported.

### 3.4. Cell and Cell Culture

The human breast cancer cell line MCF-7 was acquired from the national cell bank of Pasteur Institute of Iran (Tehran) and grown under standard cell culture conditions (37°C, 95% humidity with 5% CO_2_) in DMEM medium containing 10% FBS, 100 U/mL of penicillin and 100 μg/mL of streptomycin. The culture medium within the flask was supplanted by a fresh medium every 2-3 each other days. Assays were done using the same growth conditions. Previous to each assay, cell counts and viability were determined by trypan blue assay and hemocytometer. Only cultures displaying > 95% viability were used for experiments. All assays were performed at least with triplicates three times.

#### 3.4.1. Cell Viability (MTT Assay)

Cells were harvested with trypsin/EDTA and counted using the trypan blue dye exclusion method (17). MTT assay was used to evaluate metabolic activity. Almost 5000 cells per well were seeded in 96-well. 24 h after seeding, the cells were then treated with culture medium (control) and five different concentrations of each HDCCD (6a-j) (0.001, 0.01, 0.1, 1, and 10 mg/L) and incubated at standard cell culture conditions for 24 h again. The desired concentrations were made from 1000 mg/L stoke that drug was dissolved in DMSO. The next day MTT solution (20 μL, 0.5%) was added to each treated and non-treated well and incubated at standard cell culture conditions again for four h. The medium was then poured out, and solubilization of the crystal formazan dye was done in 200 μL DMSO. The Bio-Tek Powerwave X microplate reader (BioTek Instruments) was applied to read the absorbance at 570 nm.

The treatment-induced cell death was quantified by lactate dehydrogenase (LDH) -Cytotoxicity Assay Kit II (Source Bioscience) (18). A 96-well plate was filed with culture media on the cells (10 μL) followed by adding LDH reaction mix (100 μL) to each well. The plate was maintained at room temperature for 30 - 60 min and the microplate reader was used to read the absorbance. For good absorption, the 650 nm standard absorbance was subtracted from the 450 nm absorbance reading. Then, the cytotoxicity percentage was calculated by the following formula:

Cytotoxicity (%) = sample (OD) - low control (OD)/high control (OD) - low control (OD) × 100

Where: Low control was un-treated cell (control), and high control was treated cells with Triton X 1%

#### 3.4.2. Nitrosative and Oxidative Stress Activities

The nitric oxide (NO) and malondialdehyde (MDA) biomarkers of the exposed cells to HDCCD (6a-j) were evaluated on the cells supernatant and culture medium after 24 h incubation. To screen the effects of HDCCD (6a-j) on the nitrite concentration, 5 × 10^3^ cells were exposed at IC_50_ concentration that was earnt from MTT assay. The next day, 100 µL of cell supernatant and culture media on the cell were collected separately and incubated with 50 µL of 2% w/v sulfanilamide in 10% v/v o-phosphoric acid for 15 min at room temperature. After that 50 µL of 0.2% w/v of N-(1-naphthyl)-ethylenediamine dihydrochloride was added and left to incubate for an additional 15 min. The absorbance of the samples was determined at 570 nm by a microplate reader, and the quantification of nitrite in the sample was standardized with 0 - 100 mmol/L of nitrite concentration (NaNO_2_) (19). Also, lipid peroxidation was determined on the exposed cells to the HDCCD (6a-j) (20). In this part, the MDA content of the cell supernatant and culture media on the cells was measured using TBA reaction. 500 µL of the cell supernatant and culture media of the cells were mixed with 3 mL orthophosphoric acid (1% v/v) finely, and then TBA (2 mL; 6.7 g/L) was added to each one. Samples were heated at 100°C for 45 min, then chilled on ice, and centrifuged at 3000 rpm for 10 min. The absorbance of the supernatant was read at 532 nm, and the MDA level was determined according to the relative standard curve.

#### 3.4.3. Apoptosis Assay

To quantify apoptotic death of MCF-7 cells treated at IC_50_ concentration of the HDCCD (6g and 6d) and non-treated cells, Annexin V-FITC apoptosis detection kit was used (21). Cells (1 × 10^5^ per well) were seeded and kept under standard growth conditions for 24 h and then were exposed to the IC_50_ concentration and incubated for another 24 h. Then the exposed cells and non-exposed cells (control) were painted according to the protocol provided by the manufacturer, and the quantitative analysis for apoptosis was performed by flow cytometry.

#### 3.4.4. Molecular Docking Study

The AutoDock 4.2 (ADT) program was used for molecular docking of compounds (6a-j) into the of Cyclin-dependent kinase 6 3D X-ray structure (PDB code: 4EZ5). Construction of the 3D structures of the compounds was done, and the Open Babel software minimized the energy. The protein complex crystal structures were obtained from the RCSB protein data bank (http://www.rcsb.org). All ligands and bound water molecules were removed from the protein, and proteins were added with polar hydrogens. Kollman and Gasteiger-Hückel charges were used for the proteins and ligands, respectively. AutoDock 4.2 software performed molecular docking of all ten compounds. According to the cognate ligand, the grid box was centered with grid spacing of 0.375 Å and dimensions of 60 × 60 × 60. Genetic algorithm factors were set to 2,500,000 energy evaluations, 100 runs, and 150 population size. Cluster assessment was performed on the docked findings using an RMSD tolerance of 2 Å.

## 4. Results

**Figure 2.**
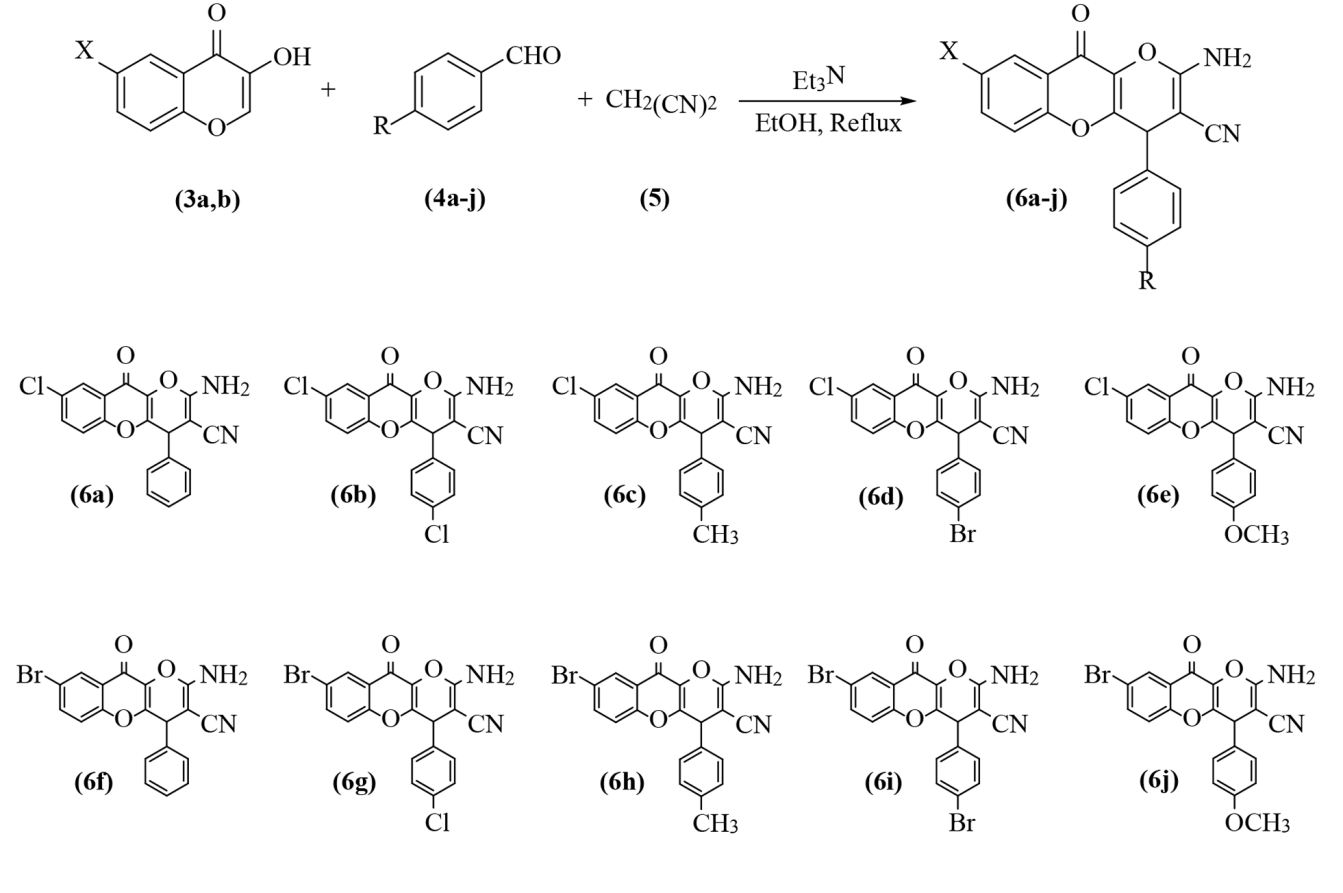
Synthesis of halogenated dihydropyrano[3,2-*b*]chromene-3-carbonitrile derivatives (6a-j)

A new HDCCD (6a-j) was synthesized according to Figures 1 and 2. The condensation of 5'-chloro- or 5'-bromo-2'-hydroxyacetophenone (1a, b) with N,N-dimethylformamide-dimethylacetal (DMFDMA) was irradiated under microwave conditions, then refluxed with concentrated HCl. This reaction led to the production of 6-chloro- and 6-bromo-chromone derivatives (2a, b), which formed epoxy chromones upon treatment with H_2_O_2_/NaOH in methylene chloride. This undergoes ring opening with concentrated HCl afforded 6-chloro- and 6-bromo-3-hydroxychromones (3a, b) in good yields (14).

Following our previous works on multicomponent reactions to reach potentially bioactive scaffolds (22-25), we carried out a new one-pot three-component reaction for the synthesis of 8-halopyrano[3,2-*b*]chromen-10(4*H*)-one derivative; (6a-j) the process includes 6-chloro- and 6-bromo-3-hydroxychromones (3a, b), aromatic aldehydes (4a-j), and malononitrile (5) in the presence of three drops of Et_3_N in ethanol as the solvent and using reflux conditions. After completion of the reaction, the crude product was purified by recrystallization, and a series of new 8-halo-4,10-dihydropyrano[3,2-*b*]chromene-3-carbonitrile derivatives (6a-j) were produced in 85-93% yields (Figure 2).

### 4.1. Ferric Reducing Antioxidant Power

**Figure 3.**
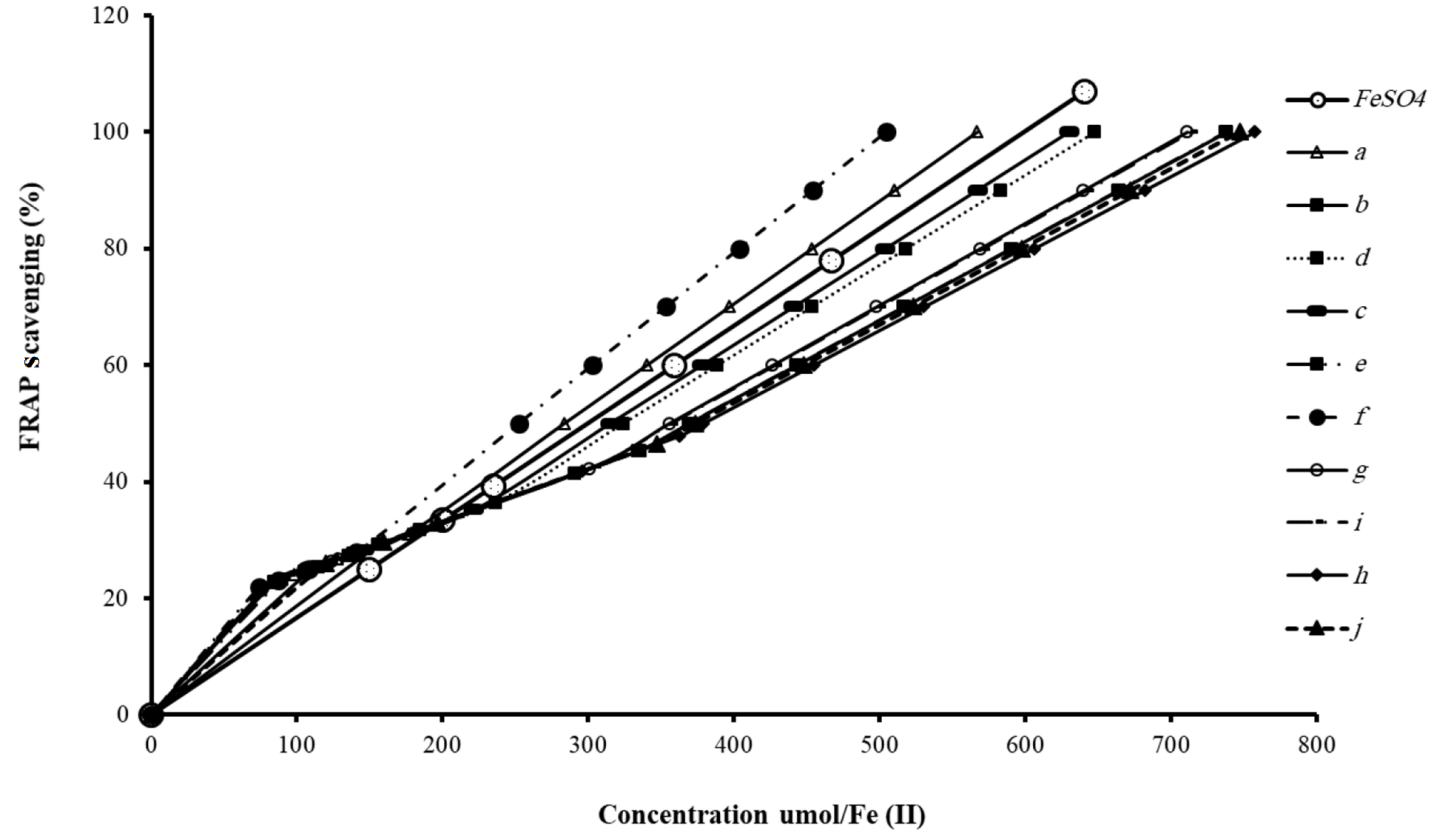
The reductive potency of the halogenated dihydropyrano[3,2-*b*]chromene-3-carbonitrile derivatives (HDCCD) (6a-j) compared to the working standard at different concentrations of 100 - 1000 umol/Fe (II)

Figure 3 shows the reductive potency of the HDCCD (6a-j) compared to the working standard at different concentrations. Results illustrated that all of the HDCCD (6a-j) had a dose-response relationship; their reducing capacity increased by increasing concentration. This increase was notable at higher concentrations. 6a and 6f showed an antioxidant capacity higher than the working standard (FeSO_4_).

Antioxidant activity with IC_50_ values of HDCCD (6a-j) was measured by FRAP radical scavenging assay (Table 1). Data showed that 6f and 6h with IC_50_ values of 252.09 and 378.8 had the highest and lowest antioxidant properties, respectively.

**Table 1.** IC_50_ Values of FRAP Scavenging Effect of the HDCCD (6a-j) (µmol/Fe (II))

HDCCD (6a-j) and Standard (FeSO_4_)	IC_50_ (FRAP)
**FeSO**_**4**_	299.40
**6a**	283.32
**6b**	369.07
**6c**	315.03
**6d**	323.59
**6e**	368.65
**6f**	252.09
**6g**	355.4
**6h**	378.8
**6i**	356.81
**6j**	373.48

Abbreviations: HDCCD, halogenated dihydropyrano[3,2-*b*]chromene-3-carbonitrile derivatives; FRAP, ferric reducing antioxidant power assay.

### 4.2. Cytotoxicity Assay

The cytotoxic effect of the HDCCD (6a-j) on MCF-7 cells was determined by MTT assay. Lactate dehydrogenase concentration of the cells exposed to the HDCCD IC_50_ concentration was also evaluated (Table 2). According to what was observed, 6a and 6f showed high IC_50_ of 13 and 8 mg/L, while IC_50_ amounts of the 6d, 6g, and 6c were low; 0.50, 0.70, and 0.90 mg/L, respectively.

**Table 2.** IC_50_ Concentration (mg/L) and LDH (U/mL) of the HDCCD (6a-j) on the MCF-7 Breast Cancer Cell Lines

HDCCD (6a-j)	IC_50_ Concentration (mg/L)	LDH (U/mL)
**6a**	13.00	0.38
**6b**	6.00	0.00
**6c**	0.90	0.00
**6d**	0.50	0.00
**6e**	5.20	0.39
**6f**	8.00	0.41
**6g**	0.70	0.44
**6h**	4.30	0.41
**6i**	5.70	0.37
**6j**	3.40	0.00
**Doxorubicin**	0.417	

Abbreviations: LDH, lactate dehydrogenase; HDCCD, halogenated dihydropyrano[3,2-*b*]chromene-3-carbonitrile derivatives.

**Figure 4.**
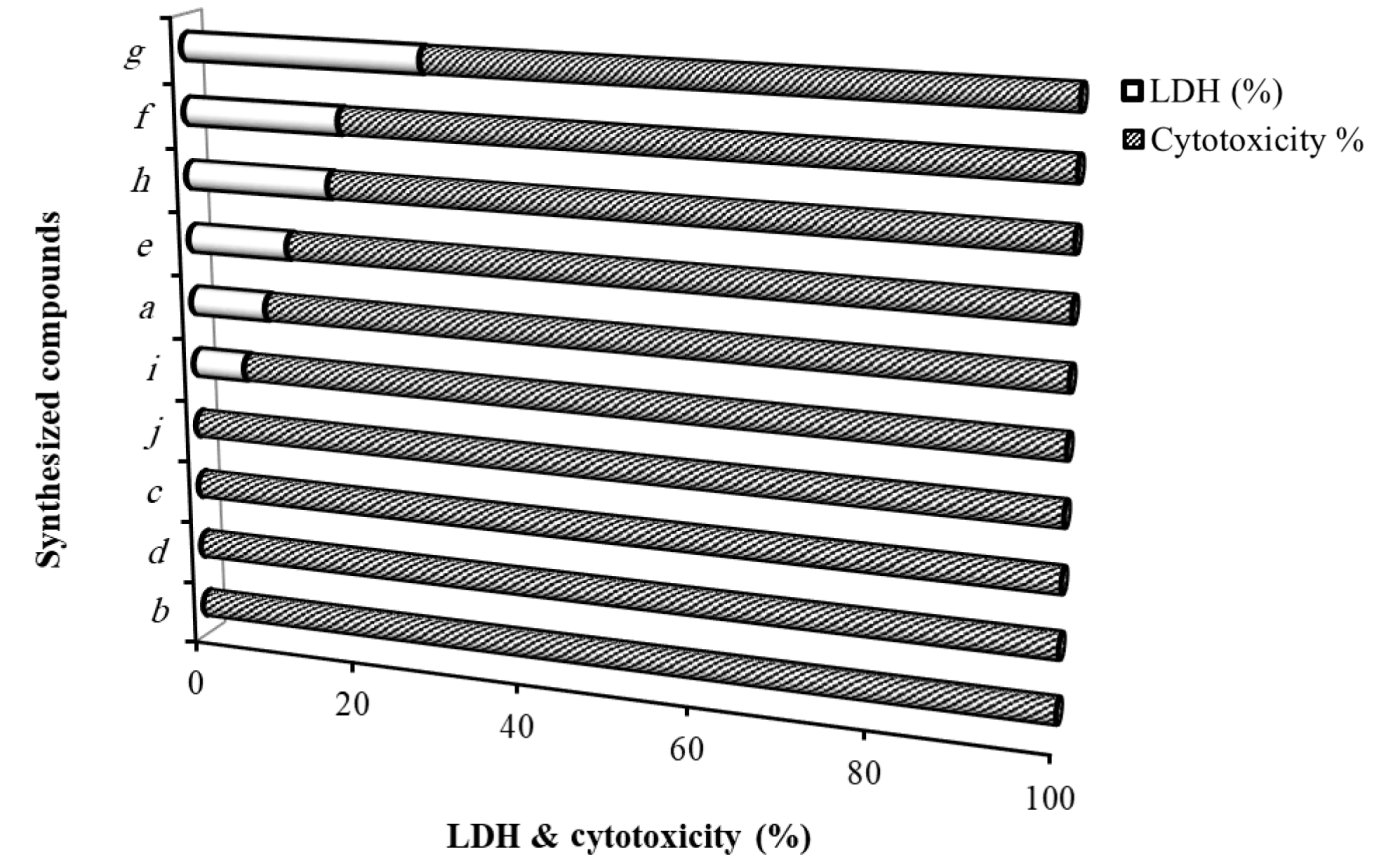
The cytotoxicity and lactate dehydrogenase (LDH) (%) on the MCF-7 cells exposed to the halogenated dihydropyrano[3,2-*b*]chromene-3-carbonitrile derivatives (HDCCD) (6a-j) compounds

The cytotoxicity and LDH (%) of the HDCCD (6a-j) are illustrated in Figure 4. Most of the HDCCD (6a-j) showed cytotoxic effects on the cells. The LDH concentration secreted by cells exposed to 6b, 6c, 6d, and 6j was zero while the maximum amount of the LDH (%) was seen in the cells treated by 6g (29 %).

### 4.3. Nitrosative and Oxidative Stress Activities

Nitrite concentration and lipid peroxidation (MDA) of the cells' supernatant and culture medium were measured. Data presented that all of the HDCCD (6a-j) increased the nitrite concentration of the exposed cells except for 6e, 6g, and 6i. Nitrite concentration of the culture media on the cells was decreased compared to the control; however, only 6b and 6i showed an increase (Table 3).

**Table 3.** Nitrite and MDA Concentration of the Cell Supernatant and Medium on the Cell After Exposure to the HDCCD (6a-j) Compared to the Non-exposed Cell (Control)

HDCCD (6a-j)	Nitrite (µM/L)		MDA (µM/mL)	
	Cell	Medium	Cell	Medium
**6a**	23 ± 1.0	32 ± 0.5	0.50 ± 0.2	2.91 ± 1.0
**6b**	37 ± 1.0	51 ± 1.0	0.30 ± 0.1	3.93 ± 1.0
**6c**	22 ± 0.2	28 ± 1.0	0.40 ± 0.2	2.11 ± 1.0
**6d**	41 ± 0.5	30 ± 0.5	0.30 ± 0.1	3.21 ± 1.0
**6e**	16 ± 1.0	31 ± 0.5	0.11 ± 0.1	2.51 ± 0.5
**6f**	25 ± 1.0	31 ± 0.5	0.20 ± 0.2	1.95 ± 1.0
**6g**	18 ± 1.0	25 ± 0.5	0.32 ± 0.1	1.93 ± 0.5
**6h**	28 ± 1.0	29 ± 1.0	0.60 ± 0.1	2.30 ± 0.2
**6i**	17 ± 1.0	47 ± 0.5	0.57 ± 0.1	2.21 ± 0.3
**6j**	39 ± 1.0	37 ± 1.0	0.50 ± 0.2	2.32 ± 0.4
**Control**	16 ± 0.5	41 ± 0.5	0.40 ± 0.2	2.58 ± 0.5

Abbreviations: MDA, malondialdehyde; HDCCD, halogenated dihydropyrano[3,2-*b*]chromene-3-carbonitrile derivatives.

Malondialdehyde levels of the cells were kept below the range of the control by the addition of 6b, 6d, 6e, 6f, and 6g on the cells, while the addition of the 6a, 6c, 6h, 6i, and 6j increased the MDA level compared to the control. Malondialdehyde levels of the medium on the cells were decreased after adding all HDCCD (6a-j) except for 6b and 6d (Table 3).

### 4.4. Quantitative Analysis of Apoptosis by Flow Cytometry

**Figure 5.**
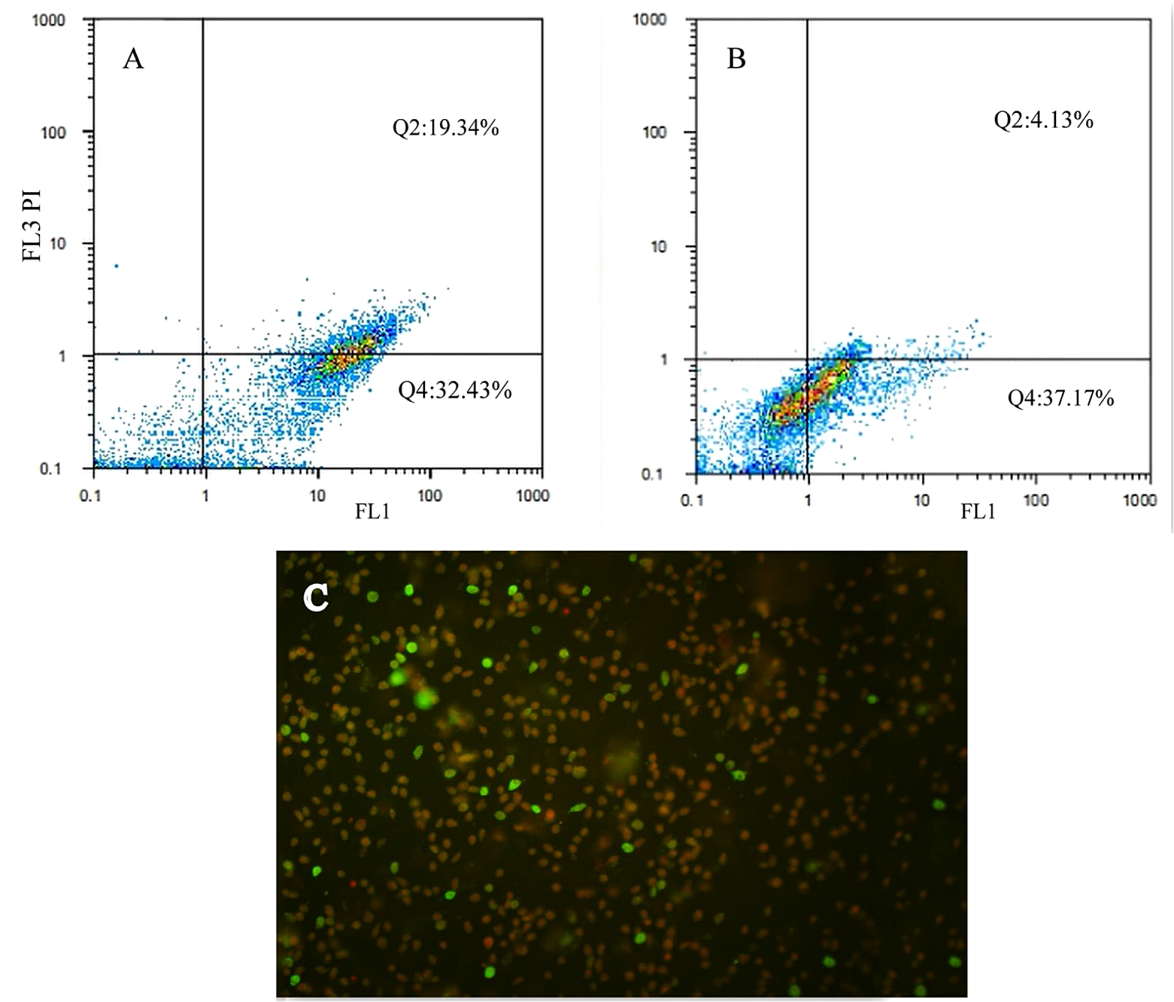
Flow cytometry analysis of the MCF-7 cells exposed to 6d and 6g at IC_50_ concentration for 24 h. A and B, quantity of apoptosis of cells exposed to 6d and 6g respectively; C, Annexin V-PI staining cell; early apoptosis (green; annexin V); late apoptosis (red and green; PI and Annexin V); necrosis (red; PI)

A flow cytometric analysis of MCF-7 cells exposed to 6d and 6g at IC_50_ concentrations 0.5 and 0.7 mg/L, respectively, was performed to analyze the quantity of apoptosis (Figure 5). Most of the cells were in the early and late apoptotic stages. A few were in the necrotic stage (Figure 5C). Flow cytometry analysis confirmed about 50% apoptosis of cells when exposed to IC_50_ concentrations of 6d and 6g (Figure 5A and B).

### 4.5. Molecular Docking Study

#### 4.5.1. Docking of Synthesized Compounds to the Active Site of Cyclin-dependent Kinase 6 (PDB Code: 4EZ5)

Cyclin-dependent kinases (CDKs) play a central role in cell cycle control, apoptosis, transcription, and neuronal functions (26). It is established that natural chromenes like fisetin are inhibitors of CDKs. Moreover, in one study, it turned out that 8-chloroflavanone, which can be regarded as a substructure of our compounds 6a-f, was the most potent flavonoid against the MCF-7 cancer cell line (27). Therefore, to suggest the interaction of compounds 6a-j with Cyclin-dependent kinases, molecular docking of the synthesized compounds (6a-j) into the active site cavity of CDK6 was performed based on the CDK6 complex structure (PDB code: 4EZ5). All compounds could accommodate the active site of the enzyme, and the top two compounds (6e and 6j), in terms of free binding energy, were selected for further investigation of interactions. The cognate inhibitor in 4EZ5 ((5-[4-(dimethylamino)piperidin-1-yl]-1H-imidazo[4,5-b] pyridin-2-yl)[2-(isoquinolin-4-yl)pyridin-4-yl]methanone) was also docked as a reference (Table 4).

**Table 4.** Final Docked Energy of Compounds (6a-j)

HDCCD (6a-j)	ΔG (kcal/mol)
**6a**	-8.50
**6b**	-8.89
**6c**	-8.49
**6d**	-8.94
**6e**	-9.37
**6f**	-8.60
**6g**	-8.70
**6h**	-8.62
**6i**	-8.99
**6j**	-9.43
**((5-[4-(dimethylamino)piperidin-1-yl]-1H-imidazo[4,5-b] pyridin-2-yl)[2-(isoquinolin-4-yl)pyridin-4-yl]methanone**	-12.51

Abbreviation: HDCCD, halogenated dihydropyrano[3,2-*b*]chromene-3-carbonitrile derivatives.

**Figure 6.**
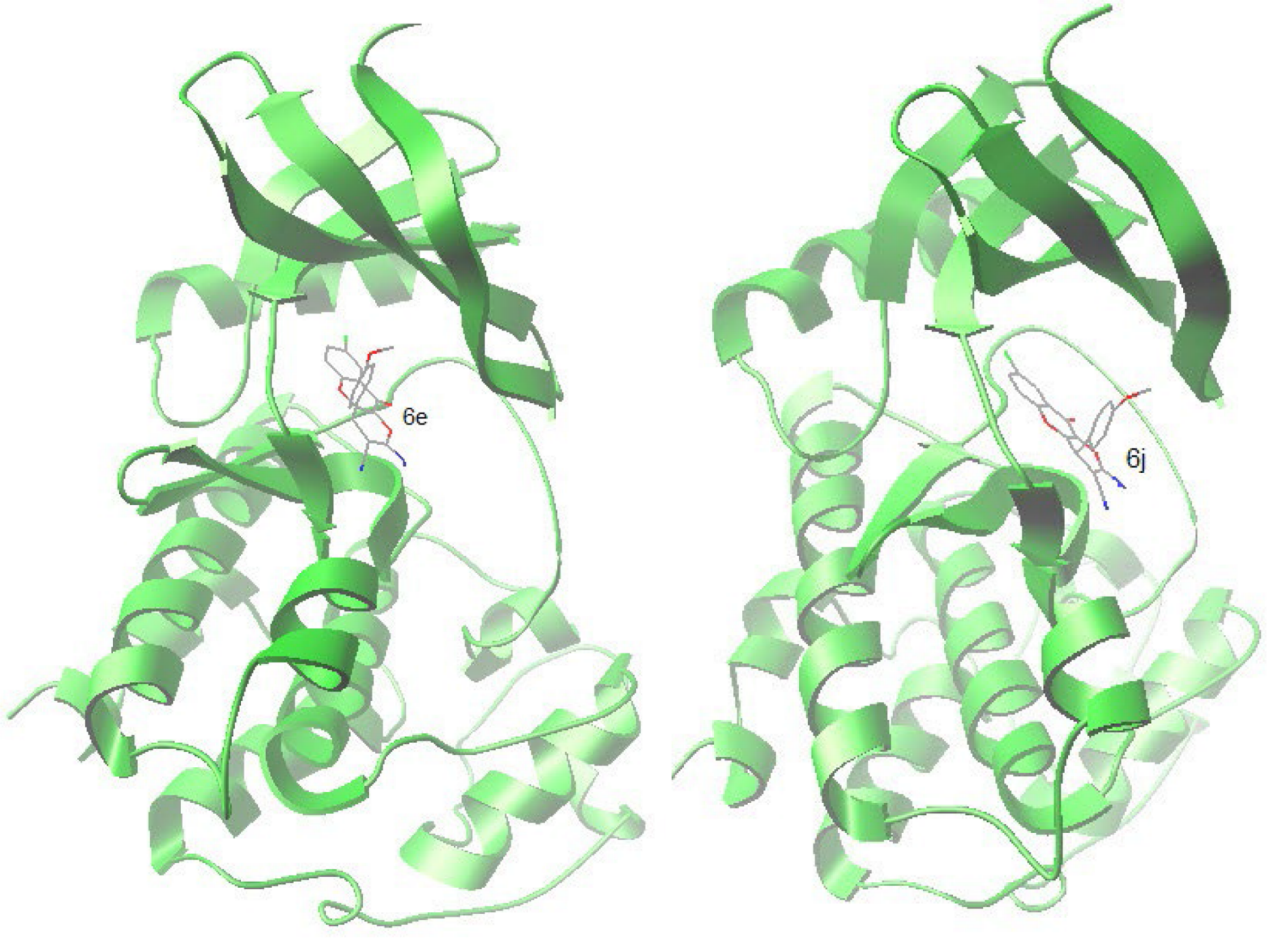
Accomodation of compounds 6e and 6j in the active site of Cyclin-dependent kinase 6 (CDK6)

**Figure 7.**
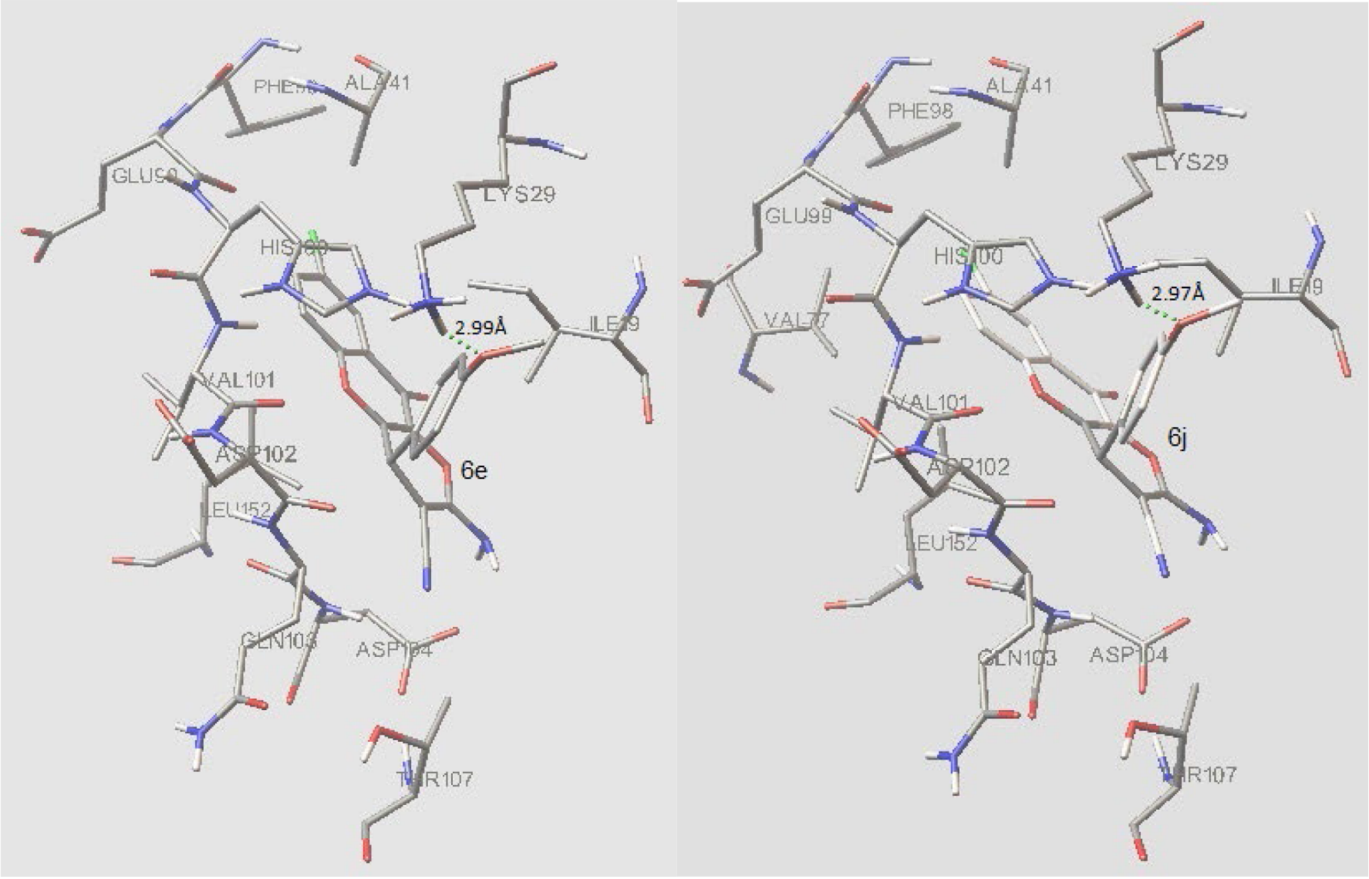
Interaction of compound 6e and 6j with active site residues of Cyclin-dependent kinase 6 (CDK6)

In the binding mode, compounds 6e and 6j were nicely bound (ΔG = -9.37 kcal/mol and -9.43 respectively) to the CDK6 active site (Figure 6) via one hydrogen bond together with multiple hydrophobic interactions (Figure 7). The methoxy oxygen of 6e formed hydrogen bond with the hydrogen of NH_2_ of Lys 29 (bond length: Lys29 N-H...O 2.99 Å) and for the compound 6j, there is a hydrogen bond between the oxygen of methoxy and hydrogen of NH_2_ of Lys 29 (bond length: Lys29 N-H...O 2.97 Å).

Both 6e and 6j form hydrogen bond interaction with LYS29 of the CDK6, while the co-crystalized inhibitor has 2 hydrogen bonds via LYS43 and VAL101 with CDK6. The following residues are important as they are present in interaction with both 6e and 6j as well as co-crystallized inhibitor; Ile 19, Lys 29, Ala41, Val77, His100, Val101, Leu152. These residues plus Lys43, Val37, Gly20, Ala 162, and Gln 149, form the active site of the enzyme.

## 5. Discussion

This study investigated the pharmaceutical characteristics of the HDCCD (6a-j) and their antioxidant, cytotoxic, stress oxidative activities, and apoptotic effects on the MCF-7 cell line. In order to evaluate the antioxidant activity, FRAP assay was performed by measuring the ability to reduce ferric (III) ions to ferrous (II) ions. Results confirmed that all the HDCCD (6a-j) showed antioxidant activity compared to the reference antioxidant (FeSO_4_). This means that all of the HDCCD (6a-j) can inhibit oxidation. It is known that oxidation is a chemical reaction that can produce free radicals and trigger chain reactions, which damage organisms. According to their chemical characteristics, the HDCCD (6a-j) belong to attractive chromene compounds because of their interesting biological activity. The chromene compound's primary backbones constitute polyphenols that act as reducing agents and antioxidants by the hydrogen-donating property of their hydroxyl groups. Therefore, the high antioxidant activity of the HDCCD (6a-j) in this study could refer to their chemical structure. One of the most important antioxidant structures is vitame E, which belongs to chromane compounds that show potent antioxidant activity.

The cytotoxic experiments were performed to confirm the toxicity effects of the HDCCD (6a-j) by MTT and LDH assays. All of the examined materials showed quality to being toxic to cells. Also, to confirm the apoptotic effects of the HDCCD (6a-j), LDH enzyme was measured on the cells exposed at IC_50_ concentration. According to the results, all of the HDCCD (6b, 6d, 6c, 6j) were safe and did not damage the cell membrane. However, 6a, 6e, 6f, 6g, 6h, and 6i showed a disturbing effect on the cell membrane and secreted LDH enzyme. Lipophilic nature of the chromene compounds (6a-j) causes them to easily cross the cell membrane, so LDH leakage is considerably low (Figure 2). Although one can evaluate the cytotoxicity of the compounds on the normal cells for better judgment, in this experiment, we only claimed that our compounds affect the cancerous cell line, MCF-7. But we decide to consider the cytotoxicity evaluation in our future studies.

Nitrosative and oxidative stress on the cell supernatant and the medium on the cell was assayed, and most of the HDCCD (6a-j) showed protective effects and changed the levels of NO and MDA in the medium on the cell. It is probable that the phenolic character of the chromene core is responsible for the radical scavenging activity through the formation of stable radicals. Cell supernatant did not show a notable difference in NO and MDA levels in the treatment and control groups. The NO and MDA levels were seen at high concentrations in some cell lines. However, as NO and MDA levels did not change notably, the cytotoxic effect of HDCCD (6a-j) may not be due to nitrosative or oxidative stress.

In this study, to recognize the apoptotic effects of the HDCCD (6a-j) on the MCF-7 cell line, a flow cytometry assay was conducted for 6d and 6g at IC_50_ concentration. The reason for choosing 6d and 6g was due to their stronger MTT results. As shown in Figure 4, 6d and 6g showed 100 and 70% toxicity, respectively. Flow cytometry assay showed near 50% apoptosis (early and late) at IC_50_ concentration, which was in agreement with MTT and LDH assay. Also, staining cells under a fluorescence microscope confirmed that most cells were in apoptotic status. From the structure-activity relationship point of view, it can be inferred that the presence of a group on the para position of the aromatic ring significantly increases the cytotoxicity of the compound. This may be attributed to the presence of a lipophilic pocket in the receptor, which leads to a better accommodation of 6b, 6c, 6d, 6e, 6g, 6h, 6i, and 6j in the active site. Although there is no clear-cut relationship between ΔG of these compounds and their IC_50_, they all accommodate well in the active site of the enzyme with a subtle difference. It is also revealed that 6a and 6f that lack substitution on the para position, have weaker interaction with the receptor, and lesser cytotoxicity compared to others (IC_50_: 13, 8 mg/L respectively).

The molecular docking study results and the biological assay data suggest that compound 6a-j can be a potential inhibitor of CDK6, but the further molecular investigation may be needed for complete confirmation. The result of this work might be helpful in the design and synthesis of novel chromene-based inhibitors with stronger activities.

## Data Availability

The data presented in this study are uploaded during submission as a supplementary file and are openly available for readers upon request.
